# Global optimization of an encapsulated Si/SiO$_2$ L3 cavity with a 43 million quality factor

**DOI:** 10.1038/s41598-021-89410-1

**Published:** 2021-05-12

**Authors:** J. P. Vasco, V. Savona

**Affiliations:** Institute of Physics, École Polytechnique Fédérale de Lausanne (EPFL), CH-1015 Lausanne, Switzerland

**Keywords:** Nanocavities, Photonic crystals

## Abstract

We optimize a silica-encapsulated silicon L3 photonic crystal cavity for ultra-high quality factor by means of a global optimization strategy, where the closest holes surrounding the cavity are varied to minimize out-of-plane losses. We find an optimal value of $Q_c=4.33\times 10^7$, which is predicted to be in the 2 million regime in presence of structural imperfections compatible with state-of-the-art silicon fabrication tolerances.

## Introduction

Photonic crystal (PC) slab cavities have been focus of intense research during the last two decades due to their unique properties to efficiently confine light at length scales close to the diffraction limit, and extremely low loss rates^1,2^. These features have allowed to study a wide variety of classical and quantum phenomena, where the linear and non-linear interactions between light and matter are effectively enhanced in the cavity region^3–20^. Broadly speaking, the strength of this enhancement grows with the local density of electromagnetic states, which is proportional to the quality factor of the cavity mode $Q_c$, and inversely proportional to its mode volume *V*^21–23^. Hence, massive efforts have been directed toward the optimization of these figures of merit in order to reach the desired functionality of the photonic device^24–29^. Particularly, silicon-based cavities have attracted very much attention because of their natural compatibility with CMOS technologies and negligible material losses at telecom wavelengths, allowing the integration with optoelectronic devices in a single chip^30^. While free-standing silicon PC slabs offer an excellent platform to build ultralow loss cavities^27,31,32^, silica (SiO$_2$) encapsulation improves the mechanical stability and thermal dissipation of the system^33^, while mitigating additional loss channels coming from the etching of air holes in the silicon^34^. Nevertheless, high quality factors are challenging in such encapsulated structures given the low refractive index contrast between the two materials.

In this work, we employ a global optimization approach to maximize the quality factor of a Si/SiO$_2$ L3 PC cavity. Since it was proposed for first time by Y. Akahane et al.^2^, the L3 cavity stands out as the paradigm of high-Q cavity with very small spatial footprint and small mode volume close to diffraction limit^24,35^. The resonant mode of the L3 cavity has Bloch components mostly lying below the radiative light cone, similarly to many other geometries of PC cavities. This configuration is well suited for optimization of the quality factor, as the small Bloch components still above the light cone—responsible for radiative losses—can be suppressed by small geometric tweaks of the cavity surroundings. For this reason, the L3 cavity has been the model of many recent optimization strategies, both with conventional Maxwell simulators and more recently with the help of deep learning^36^. Here, we find an optimal quality factor of $Q_c=4.33\times 10^7$, which corresponds to a remarkable low out-of-plane losses given the short length of the cavity. Our results set a new record for the L3 paradigm and open the way to a new class of highly efficient and ultra-compact optical devices for linear and non-linear applications in classical and quantum photonics.

**Figure 1:**
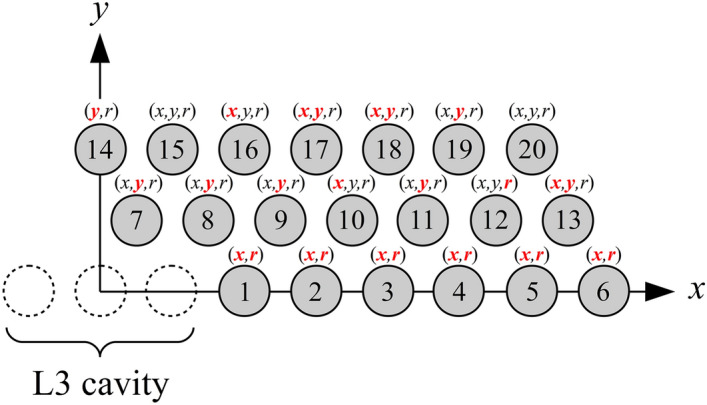
Schematic representation of the closest holes surrounding the L3 cavity, in the first quadrant, which are considered in the global optimization procedure. Mirror symmetry with respect to $x=0$ and $y=0$ is assumed, thus setting a total of 53 optimization parameters. Nevertheless, only the ones highlighted in red are found to be the most relevant to increase the fundamental mode quality factor.

## L3 cavity optimization

We consider a silica-encapsulated silicon PC slab with a hexagonal lattice of holes of radii $r=100$ nm, lattice parameter $a=390$ nm and thickness $d=220$ nm. Refractive indices of SiO$_2$ and Si are taken at telecom wavelengths, i.e., $n_{\mathrm {SiO}_2}=1.44$ and $n_{\mathrm {Si}}=3.47$, respectively. A L3 cavity is introduced by removing three holes along the $\Gamma K$ direction of the lattice. In order to optimize the quality factor $Q_c$ of its fundamental mode, we adopt a global optimization approach in which only the closest holes surrounding the cavity are varied, in size $r\rightarrow r+dr$ and position $(x,y)\rightarrow (x+dx,y+dy)$, to reduce out-of-plane losses. This technique has been extremely successful during the last few years to reach record theoretical and experimental quality factors for a wide variety of different materials and cavity geometries^20,25,27,35,37–39^. For a single optimization step, the quality factor of a given configuration must be efficiently computed. To this purpose, we use the guided mode expansion (GME) method^40^ which, for PC slab structures, has proven to be as predictive as first-principle commercial Maxwell simulators like FDTD, while being several orders of magnitude faster (see methods). This allows to simulate hundreds of thousands configurations as needed by most optimization algorithms. In addition to a Maxwell solver, one needs an efficient global optimization algorithm to find the global maximum of the quality factor in the complex landscape in parameter space. For this, non-gradient-based algorithms have empirically proven to be very effective in the past. One in particular – the particle swarm (PS) algorithm – was successfully used in past PC optimization works and turned out to be very effective also in the present analysis. As an additional advantage, as discussed in what follows, the PS algorithm is embarrassingly parallel and can be run on a multi-core architecture with dramatic computational advantage. We show in Fig. 1 the schematic representation of the holes to be considered in the optimization procedure, where mirror symmetry with respect to the planes $x=0$ and $y=0$ is assumed. Thereby, we end up with a total of 53 optimization parameters. However, after 1400 iterations of the PS algorithm we have noted that the most relevant parameters for increasing $Q_c$ are those highlighted in red in Fig. 1. This preliminary analysis allowed us to reduce the dimension of the optimization parameter space from 53 to 27, and decrease the number of function evaluations required by the algorithm to converge. We summarize in Table 1 our final results where

**Table 1:** Summary of the main linear and non-linear figures of merit of the non-optimized and optimized Si/SiO$_2$ L3 cavities.

Si/SiO$_2$ – L3 cavity	*f* (Thz)	$Q_c$	$V_l$ $(\lambda /n_{\mathrm{Si}})^3$	$V_{nl} {(\lambda/{n}_{SI})}^{3}$	$Q_{c} /N_{I} (n_{{SI}} /\lambda )^{3}$	$Q_{c}^{2} /V_{{nl}}^{2} (n_{{SI}} /\lambda )^{6}$
Non-optimized	195.2	$1.33\times 10^3$	0.67	3.25	$1.99\times 10^3$	$1.68\times 10^{5}$
Optimized	191.2	$4.33\times 10^7$	1.75	7.47	$2.47\times 10^7$	$3.36\times 10^{13}$

1 $$\begin{aligned} V_l=\frac{\int \epsilon (\mathbf {r})|\mathbf {E}(\mathbf {r})|^2d\mathbf {r}}{\text{ Max }\left\{ \epsilon (\mathbf {r})|\mathbf {E}(\mathbf {r})|^2 \right\} }, \end{aligned}$$

is the linear mode volume and

2 $$\begin{aligned} V_{nl}=\frac{\left[ \int \epsilon (\mathbf {r})|\mathbf {E}(\mathbf {r})|^2d\mathbf {r}\right] ^2}{\int \epsilon ^2(\mathbf {r})|\mathbf {E}(\mathbf {r})|^4d\mathbf {r}}, \end{aligned}$$

is the non-linear one^41^, with $\epsilon (\mathbf {r})$ representing the dielectric function of the system and $\mathbf {E}(\mathbf {r})$ the electric field of the cavity mode. An optimal solution of $Q_c=4.33\times 10^7$ (computed with FDTD^42^) is found after 806200 function evaluations, leading to an improvement of four orders of magnitude with respect to the non-optimized cavity. While this theoretical quality factor is around 50% of the record value achieved in glass-clad hetero-structured cavities^43^, it corresponds to the largest reported for the encapsulated L3 cavity. Moreover, the short-length mode confinement provided by the L3 geometry is more convenient for applications in ultra-compact devices. It is noteworthy that, $Q_c$ is maximized at the expense of the linear and non-linear mode volumes, in contrast to previous optimizations of the L3 cavity^25^. Nevertheless, we still get extremely large enhancement factors $Q_c/V_l$ and $Q_c^2/V^2_{nl}$ which are in the $10^7$ and $10^{13}$ regimes, respectively. The increase of the mode volume is clearly seen in the Fig. 2, where we plot the near-field intensity distribution of the fundamental cavity mode in the middle of the slab, for the non-optimized cavity, Fig. 2(a), and the optimized one, Fig. 2(b). The holes which are actually varied are represented by magenta circles in Fig. 2(b). The optimal parameters of the cavity as well as the far-filed projection of the near-field components are reported in the Supplementary Information.

**Figure 2:**
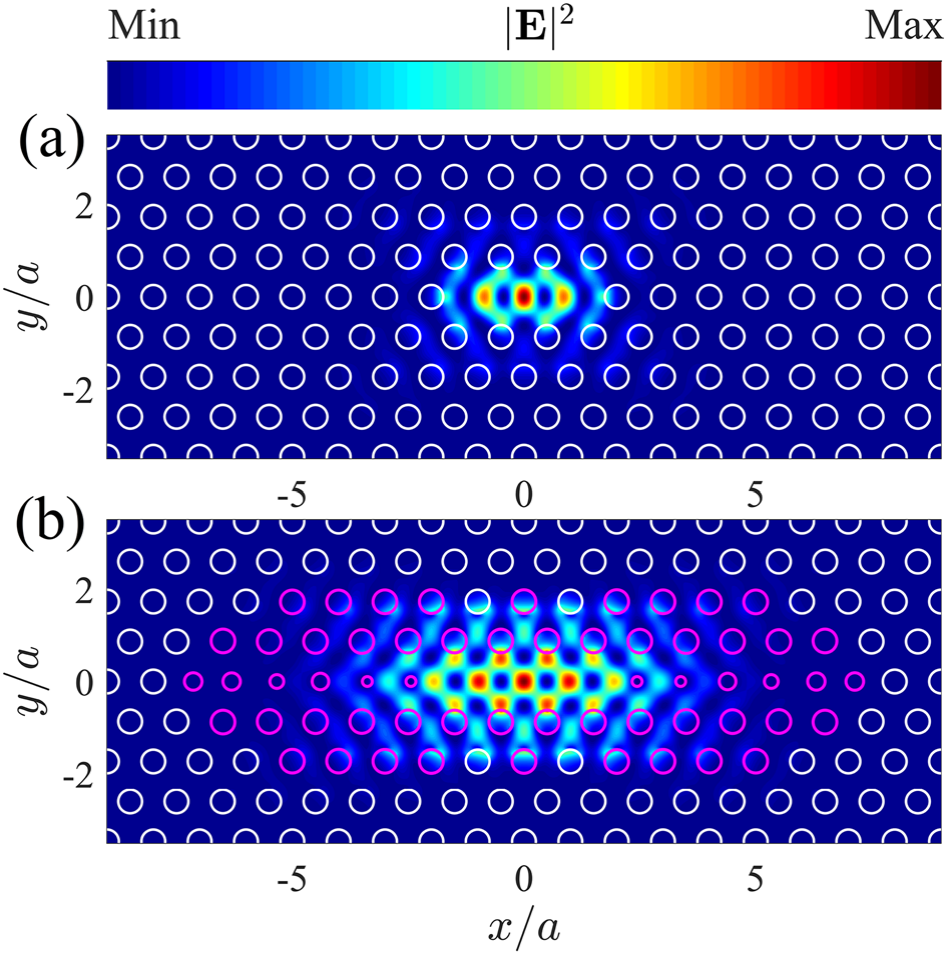
(**a**) Near-field intensity distribution of the non-optimized L3 fundamental mode cavity. (**b**) same as (a) for the optimized L3 cavity, where the holes which are actually considered in the optimization are represented by magenta circles.

The same optimization strategy can be directly applied to the air-bridge silicon L3 cavity within the same parameter space of dimension 27. For this configuration, we have obtained an FDTD quality factor $Q_c=1.91\times 10^8$. Such $Q_c$ is around 20 times larger than the previous record obtained with deep neural networks^36^, where a different set of parameters were considered to optimize the objective function. While our optimization requires a much larger number of evaluations to find the maximum of the objective function, it clearly shows that there is still considerable room for further improvement of these figures of merit by better identifying the optimization parameters. Detailed results for the Si/Air L3 cavity are given in the Supplementary Information.

## Disorder analysis

**Figure 3:**
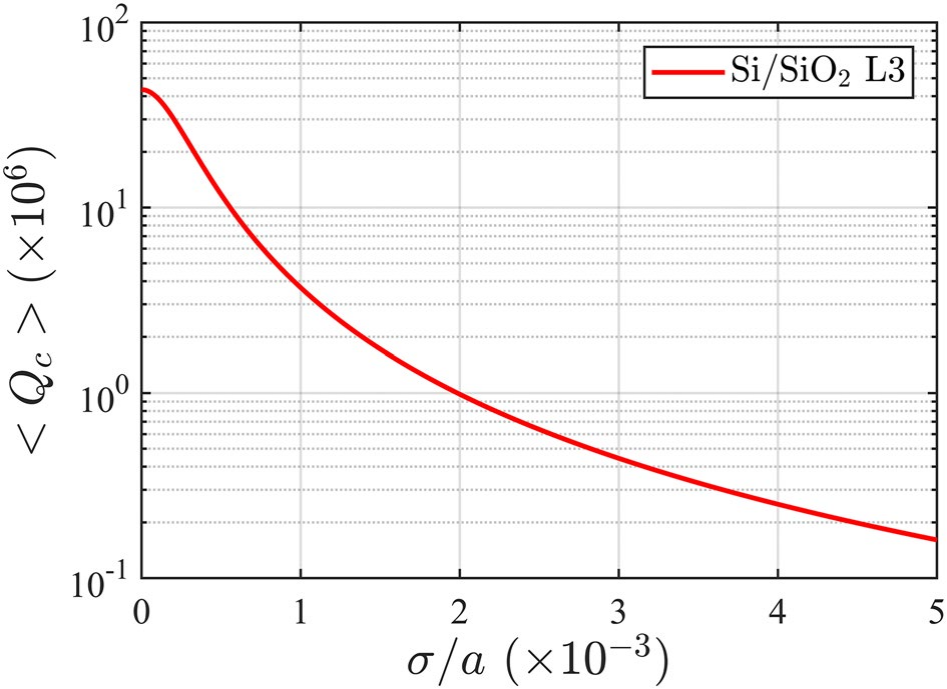
Averaged $Q_c$, computed over 100 independent disorder realizations of the optimal cavity, as a function of the disorder parameter $\sigma$.

Realistic photonic devices are always subject to a small amount of intrinsic disorder, coming from unavoidable imperfections introduced at the fabrication stage. We model such effect by considering random Gaussian fluctuations in all hole positions and radii of our PC, where the standard deviation of the Gaussian probability distribution $\sigma$ is taken as the disorder parameter^44–46^. Results of this analysis are shown in Fig. 3, where the averaged cavity quality factor $\langle Q_c\rangle$, computed over 100 independent disorder realization of the system, is plotted as a function of $\sigma$. Typical tolerances in silicon state-of-the-art fabrication techniques range between $\sigma =0.001a$ and $\sigma =0.002a$^31,47^, which leads to an averaged $Q_c$ in the 2 million regime. Previous measured *Q* values on 2D PC slab cavities with low index claddings range from $0.6\times 10^6$ (silica cladding)^48^ to $1\times 10^6$ (glass cladding)^43^, with estimated disorder magnitudes larger than $\sigma =0.002a$. However, the confinement mechanism is based on hetero-structured PCs waveguides or effective photonic potentials which usually require a long-length cavity region, i.e., more than 20 periods of the underlying photonic lattice.

## Conclusions

In conclusion, we have optimized a silica-encapsulated silicon L3 cavity by means of a global optimization strategy, where the closest holes surrounding the cavity are varied to decrease out-of-plane losses. We have found a value of $Q_c=4.33\times 10^7$, corresponding to outstanding result given the short-length of the confinement region. To better relate our optimal design to realistic samples, we have also studied the effects of intrinsic disorder. When considering typical tolerances in modern fabrication techniques, the averaged quality factor of the optimized cavity remains in the 2 million regime, which is comparable to previous measurements in fully embedded hetero-structured and photonic potential based designs. Apart from setting a new record for the encapsulated L3 cavity, our results open the way to a new class of optimized designs in low-index-contrast materials, such as AlN, GaN or Si$_3$N$_4$, holding great promise for nonlinear optical enhancement, sensing, and solid-state quantum optics.

## Methods

### The guided mode expansion

We employ the guided mode expansion method (GME)^40^ as our main PC solver for estimating the cavity mode frequencies and quality factors at the optimization stage. The expansion was carried out in a supercell of dimensions $20a\times 7\sqrt{3}a$ with 8517 plane waves and one TE guided mode. During the optimization procedure we mainly focus on the growing direction of $Q_c$ within the high-dimensional space of parameters. Therefore, only one *k* point is needed (in the Brillouin zone of the supercell) to compute the real $\mathfrak {R}\{\omega _c\}$ and imaginary $\mathfrak {I}\{\omega _c\}$ parts of the mode frequencies $\omega _c$. The cavity quality factor, associated to out-of-plane losses, is thus defined as^40^

3 $$\begin{aligned} Q_c=\frac{\mathfrak {R}\{\omega _c\}}{2\mathfrak {I}\{\omega _c\}}. \end{aligned}$$

Note that, while a single *k* point describes the topology of the optimization space with accuracy (which is enough for the PS algorithm), it does not give the actual value of $Q_c$. This is why the GME estimation of $Q_c$ with a single *k* may differ significantly from the value computed with FDTD (see Supplementary information). In order to recover the absolute quality factor of the cavity with GME, Eq. (3) must be integrated over *k* in the Brillouin zone of the supercell.

### Particles Swarm algorithm

We have used the parallel version of the Particles Swarm (PS) algorithm available in the Global Optimization Toolbox of MATLAB. It starts from a random swarm, uniformly distributed, of 139 particles and requires 5800 iterations to converge. Every particle at each iteration corresponds to an independent evaluation of $Q_c$ with GME executed by one CPU core. The total number of cores used in the computation is 139. This results in 806200 GME simulations to find the maximum of the objective function $Q_c$. It is worth mentioning that the number of iterations can be reduced by increasing the number of particles in the swarm, which may be advantageous if more cores are employed in the optimization.

### FDTD numerical experiments

Once the optimal parameters are found by the PS optimization, we simulate the final structure with a commercial FDTD solver^42^. The cavity mode is excited with an electric dipole oriented along the dominant mode polarization $E_y$. This source was set to be a standard Gaussian pulse, centered at the cavity frequency with a narrow bandwidth of 0.88 THz. The total size of the PC structure was $86a\times 75\sqrt{3}a/2$ with the L3 cavity localized at the center of the computational cell. PML boundary conditions and mirror symmetry with respect to the planes $x=0$, $y=0$ and $z=0$ were assumed. Finally, maximum mesh steps of *a*/40 and $0.5\sqrt{3}a/40$ were considered along *x* and *y* directions, respectively, while 40 cells per wavelength with a grading factor of 1.41421 were set along the *z* direction.

## Supplementary information

Supplementary material 1 (pdf 1927 KB)
