# Supplementary material for: Global optimization of an encapsulated Si/SiO2 L3 cavity with a 43 million quality factor
Source: Sci Rep. 2021 May 12;11:10121. doi: 10.1038/s41598-021-89410-1 (PMC8115079; doi:10.1038/s41598-021-89410-1)
Supplement: Supplementary file 1 — Supplementary material 1 (pdf 1927 KB) [file 41598_2021_89410_MOESM1_ESM.pdf]

Supplementary information to:
Global optimization of an encapsulated Si/SiO$_2$ L3 cavity with a 43 million quality factor

J.P. Vasco[1,*] and V. Savona[1]

[1]Institute of Physics, École Polytechnique Fédérale de Lausanne (EPFL), CH-1015 Lausanne, Switzerland
[*]juan.vasco@epfl.ch

1 Optimal parameters of the encapsulated L3 cavity

The optimal 27 parameters of the Si/SiO$_2$ L3 cavity with an FDTD quality factor of $Q_c = 4.33 \times 10^7$ are reported in Table 1.

Table 1. Optimal parameters of the Si/SiO$_2$ L3 cavity with $Q_c = 4.33 \times 10^7$. Parameter shift is abbreviated as P. shift.

P. shift / Hole	1	2	3	4	5	6	7	8	9	10
dx/a	0.4407	0.3817	0.3936	0.3352	0.3097	0.1385	×	×	×	0.0010
dy/a	×	×	×	×	×	×	0.0109	0.0107	0.0082	×
dr/a	-0.1500	-0.1500	-0.0772	-0.1075	-0.0690	-0.0672	×	×	×	×
P. shift / Hole	11	12	13	14	15	16	17	18	19	20
dx/a	×	×	-0.0044	×	×	0.0010	0.0018	0.0017	×	×
dy/a	0.0027	×	0.0121	-0.0010	×	×	-0.0044	-0.0081	-0.0071	×
dr/a	×	0.0001	×	×	×	×	×	×	×	×

1.1 Parameter variation bounds in the PS optimization

Parameters must be bounded through the PS optimization in order to avoid extremely small holes and bridges, or non-physical solutions. We report in Table 2 the variation bounds used for the encapsulated L3 cavity optimization.

Table 2. Parameter variation bounds for the encapsulated L3 cavity. Parameter shift is abbreviated as P. shift.

P. shift / Hole	1	2	3	4	5	6	7
dx/a	[0.2,0.45]	[0.2,0.45]	[0.2,0.45]	[0.1,0.45]	[0.1,0.45]	[-0.05,0.4]	×
dy/a	×	×	×	×	×	×	[-0.06,0.06]
dr/a	[-0.15,0]	[-0.15,0.015]	[-0.15,0.015]	[-0.15,0.015]	[-0.15,0.015]	[-0.15,0.015]	×
P. shift / Hole	8	9	10	11	12	13	14
dx/a	×	×	[-0.06,0.06]	×	×	[-0.06,0.06]	×
dy/a	[-0.06,0.06]	[-0.06,0.06]	×	[-0.06,0.06]	×	[-0.06,0.06]	[-0.06,0.06]
dr/a	×	×	×	×	[-0.06,0.06]	×	×
P. shift / Hole	15	16	17	18	19	20	
dx/a	×	[-0.06,0.06]	[-0.06,0.06]	[-0.06,0.06]	×	×	
dy/a	×	×	[-0.06,0.06]	[-0.06,0.06]	[-0.06,0.06]	×	
dr/a	×	×	×	×	×	×	

1.2 Criterion to choose the 27 most important parameters

After 1400 iterations of the PS algorithm using the whole set of 53 parameters, we found that some of them are slightly varied in the optimal solution. This fact is shown in Fig. 1. In order to simplify the optimization procedure and reduce the total number of iterations required for convergence, we keep only those parameters with variations larger than $0.019a$ [horizontal red line in Fig. 1(b)], which are the 27 ones considered in the main manuscript. Note that, once these parameters are selected, the PS algorithm is started again from the beginning but in this 27-dimensional space.

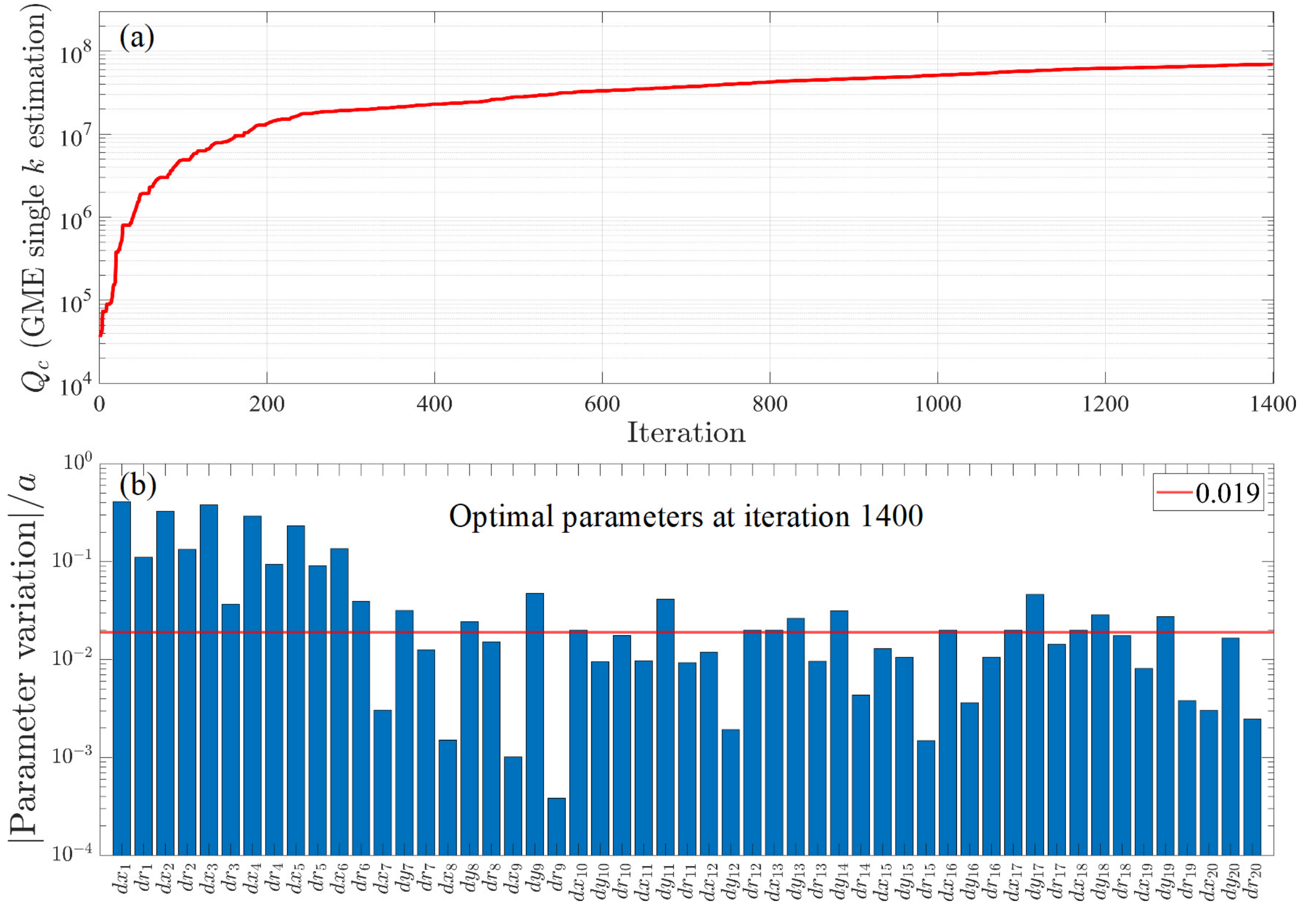

Figure 1. (a) GME single k estimation of the quality factor Q_c as a function of the iteration number when considering 53 parameters. (b) Optimal parameter variations from (a) at the iteration 1400. Only 27 parameters have variations larger than $0.019a$.

1.3 Convergence of the quality factor

We show in Fig. 2(a) the evolution of the quality factor through the optimization process. Note that the quality factor is over estimated in our single k GME computation. However, the important feature required by the PS algorithm to work properly is the Q_c landscape within the high-dimensional space. The relative error as a function of iteration number is shown in Fig. 2(b), where its average over the last 1000 iterations is found to be 2.8×10^{-5}.

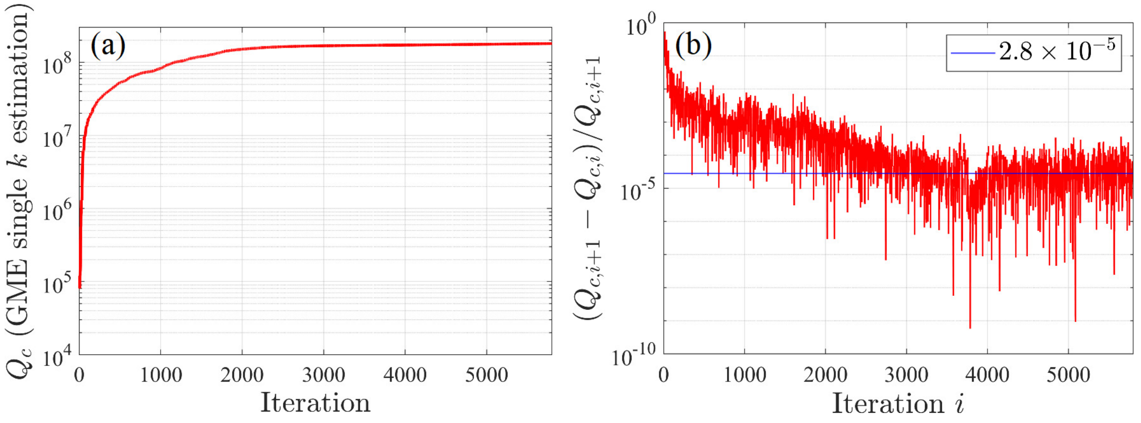

Figure 2. (a) GME single k estimation of the quality factor Q_c as a function of the iteration number when considering 27 parameters. (b) Relative error of Q_c as a function of the iteration number.

1.4 Flow chart of the PS optimization

We show in Fig. 3 the flow chart of the optimization process based on the PS algorithm to minimize the objective function f. In our case, this function is evaluated with GME and defined as $f_i = -Q_c(\overrightarrow{x_i})$, where $\overrightarrow{x_i}$ is the position of the particle i.

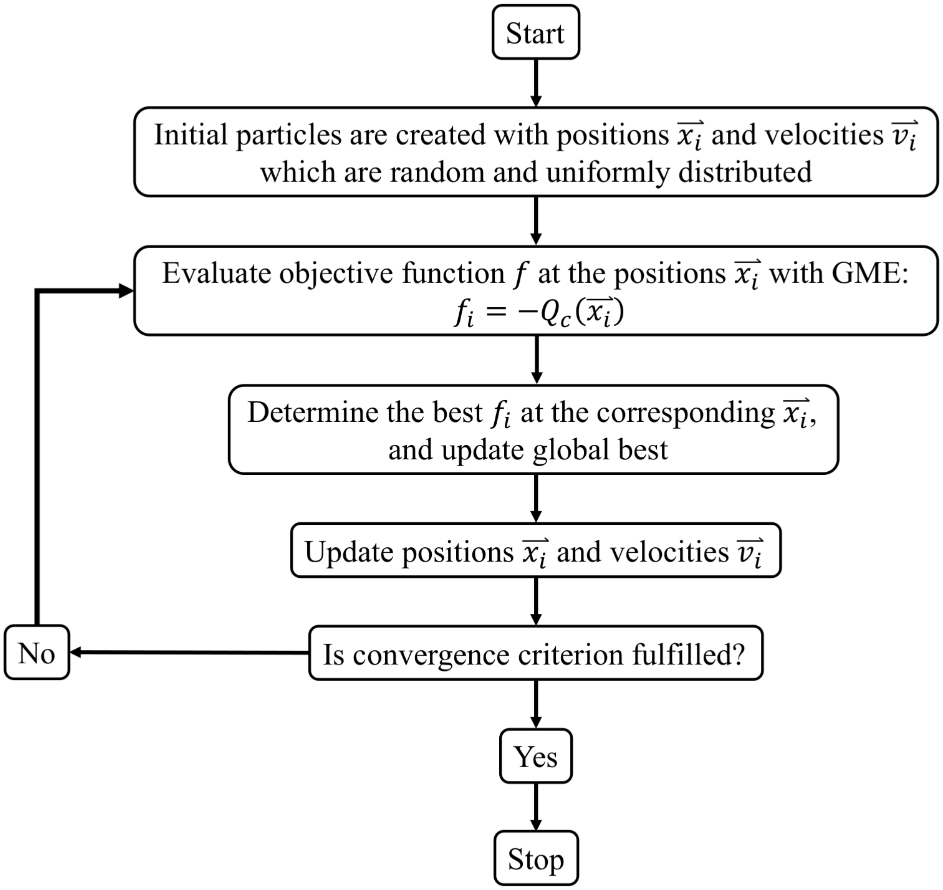

Figure 3. Flow chart of the optimization process where the objective function is evaluated with GME.

2 Fourier transform of near-field components

The far-field projections of the mode components for the non-optimized and optimized cavities, are shown in Figs. 4(a) and 4(b), respectively, in log scale. This projection is obtained through the Fourier transform of the near-field components[1], recorded in a *xy* plane localized at 90 nm above the photonic crystal surface. The dashed circle represents the region where the cavity frequency crosses the light-line. The strong reduction of the field components inside the light cone (or equivalently, above the light-line) is clearly seen for the optimized design.

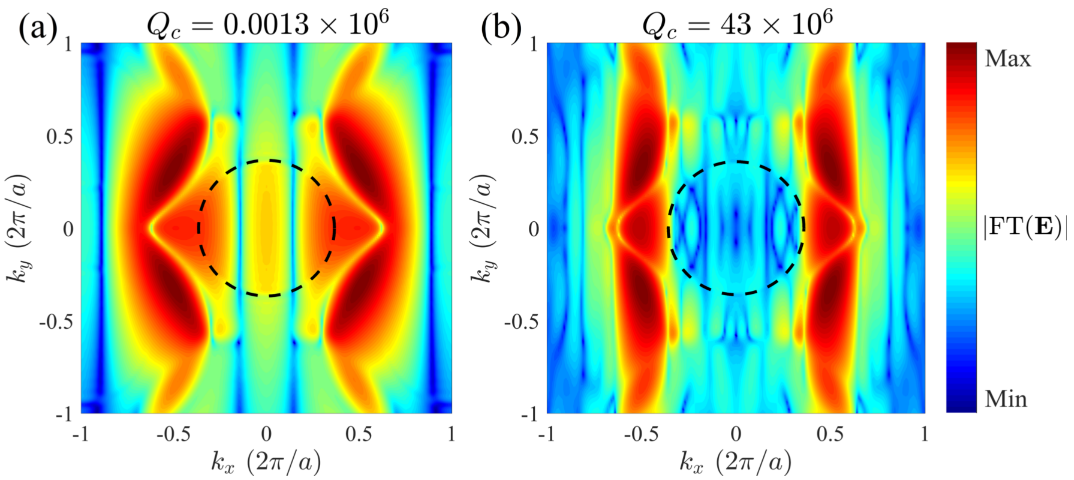

Figure 4. (a) Far-field of the non-optimized cavity. (b) same as (a) for the optimized design. The dashed circle represents the region where the cavity frequency crosses the light-line of the dielectric slab.

3 Results for the Si/Air L3 cavity

The Si/Air (air-bridge) L3 cavity is considered in a silicon PC with a hexagonal lattice of holes with radii r = 100 nm, lattice parameter a = 400 nm and slab thickness d = 220 nm. The PS optimization is carried out by considering the same 27 parameters of the Si/SiO_2 case.

3.1 Optimal figures of merit

We show in Table 3 the linear and non-linear figures of merit of both, non-optimized and optimized designs. The quality factor is improved by four orders of magnitude with a final FDTD value of $Q_c = 1.91 \times 10^8$, which is around 20 times larger than the previous best, for the silicon L3 cavity, obtained with deep learning optimization techniques[2].

Table 3. Linear and non-linear figures of merit for the non-optimized and optimized Si/Air L3 cavities.

Si/Air – L3 cavity	f (Thz)	Q_c	V_l $(\lambda/n_{\mathrm{Si}})^3$	V_{nl} $(\lambda/n_{\mathrm{Si}})^3$	Q_c/V_l $(n_{\mathrm{Si}}/\lambda)^3$	Q_c^2/V_{nl}^2 $(n_{\mathrm{Si}}/\lambda)^6$
Non-optimized	196.3	6.53×10^3	0.59	2.48	1.10×10^4	6.94×10^6
Optimized	193.6	1.91×10^8	1.07	4.30	1.78×10^8	1.97×10^{15}

3.2 Optimal parameters

The optimal parameters for the Si/Air L3 cavity with $Q_c = 1.91 \times 10^8$ are reported in Table 4

Table 4. Optimal parameters of the Si/Air L3 cavity with $Q_c = 1.91 \times 10^8$. Parameter shift is abbreviated as P. shift.

P. shift / Hole	1	2	3	4	5	6	7	8	9	10
dx/a	0.3800	0.2954	0.2000	0.4032	0.2360	0.0475	×	×	×	-0.0179
dy/a	×	×	×	×	×	×	-0.0232	-0.0157	0.0028	×
dr/a	-0.0445	-0.0174	0.0033	-0.0433	-0.1500	-0.0805	×	×	×	×
P. shift / Hole	11	12	13	14	15	16	17	18	19	20
dx/a	×	×	-0.0341	×	×	0.0040	-0.0001	-0.0059	×	×
dy/a	-0.0600	×	-0.0141	-0.0078	×	×	0.0083	-0.0114	-0.0307	×
dr/a	×	-0.0427	×	×	×	×	×	×	×	×

3.3 Disorder analysis

Figure 5 show the disorder analysis for the optimal air-bridge L3 cavity. An averaged Q_c in the 4 million regime is predicted for typical tolerances, ranging between $\sigma = 0.001a$ and $\sigma = 0.002a$, in silicon fabrication techniques[3,4].

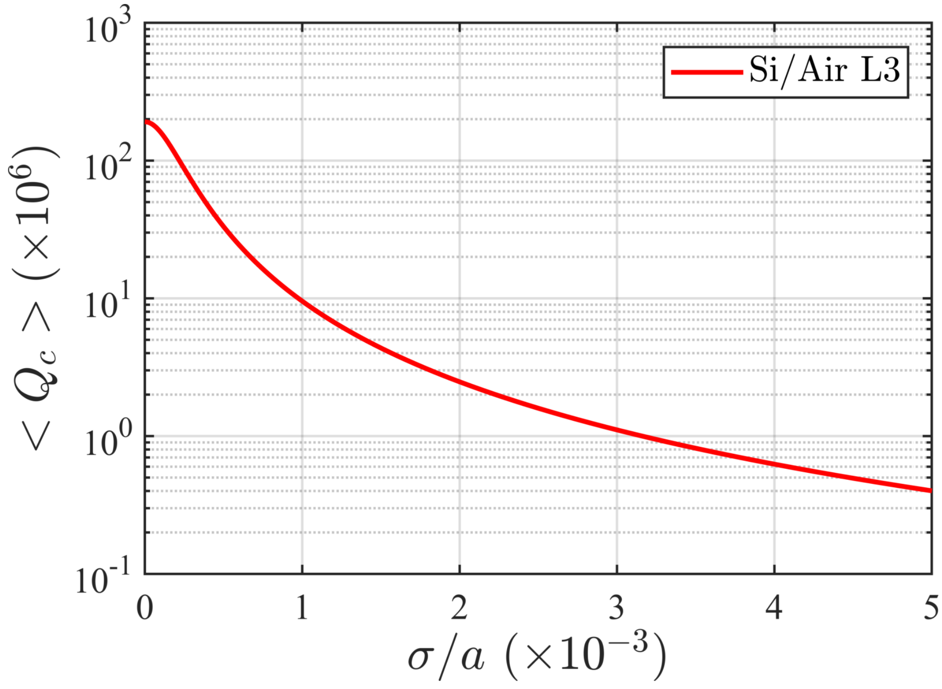

Figure 5. Averaged Q_c, computed over 100 independent disorder realizations of the optimal Si/Air cavity, as a function of the disorder parameter σ.

References

1. Vučković, J., Loncar, M., Mabuchi, H. & Scherer, A. Optimization of the q factor in photonic crystal microcavities. *IEEE J. Quantum Electron.* **38**, 850 (2002).
2. Asano, T. & Noda, S. Iterative optimization of photonic crystal nanocavity designs by using deep neural networks. *Nanophotonics* **8**, 2243 (2019).
3. Asano, T., Ochi, Y., Takahashi, Y., Kishimoto, K. & Noda, S. Photonic crystal nanocavity with a q factor exceeding eleven million. *Opt. Express* **25**, 1769 (2017).
4. Mohamed, M. S. *et al.* Influence of disorder and finite-size effects on slow light transport in extended photonic crystal coupled-cavity waveguides. *ACS Photonics* **5**, 4846 (2018).
